# Validating the Generalizability of Ophthalmic Artificial Intelligence Models on Real-World Clinical Data

**DOI:** 10.1167/tvst.12.11.8

**Published:** 2023-11-03

**Authors:** Homa Rashidisabet, Abhishek Sethi, Ponpawee Jindarak, James Edmonds, R. V. Paul Chan, Yannek I. Leiderman, Thasarat Sutabutr Vajaranant, Darvin Yi

**Affiliations:** 1 Department of Biomedical Engineering, University of Illinois Chicago, Chicago, IL, USA; 2 Artificial Intelligence in Ophthalmology (Ai-O) Center, University of Illinois Chicago, Chicago, IL, USA; 3 Illinois Eye and Ear Infirmary, Department of Ophthalmology and Visual Sciences, University of Illinois Chicago, Chicago, IL, USA

**Keywords:** generalizability, deep learning, computer-aided diagnosis, glaucoma classification, optic disc segmentation, fundus image

## Abstract

**Purpose:**

This study aims to investigate generalizability of deep learning (DL) models trained on commonly used public fundus images to an instance of real-world data (RWD) for glaucoma diagnosis.

**Methods:**

We used Illinois Eye and Ear Infirmary fundus data set as an instance of RWD in addition to six publicly available fundus data sets. We compared the performance of DL-trained models on public data and RWD for glaucoma classification and optic disc (OD) segmentation tasks. For each task, we created models trained on each data set, respectively, and each model was tested on both data sets. We further examined each model's decision-making process and learned embeddings for the glaucoma classification task.

**Results:**

Using public data for the test set, public-trained models outperformed RWD-trained models in OD segmentation and glaucoma classification with a mean intersection over union of 96.3% and mean area under the receiver operating characteristic curve of 95.0%, respectively. Using the RWD test set, the performance of public models decreased by 8.0% and 18.4% to 85.6% and 76.6% for OD segmentation and glaucoma classification tasks, respectively. RWD models outperformed public models on RWD test sets by 2.0% and 9.5%, respectively, in OD segmentation and glaucoma classification tasks.

**Conclusions:**

DL models trained on commonly used public data have limited ability to generalize to RWD for classifying glaucoma. They perform similarly to RWD models for OD segmentation.

**Translational Relevance:**

RWD is a potential solution for improving generalizability of DL models and enabling clinical translations in the care of prevalent blinding ophthalmic conditions, such as glaucoma.

## Introduction

Glaucoma is the leading cause of global irreversible blindness, affecting over 70 million people globally, and is estimated to increase up to 111.8 million by 2040.^1^^,^^2^ Early detection can prevent vision loss due to glaucoma.^1^ Recently, artificial intelligence (AI) has shown promising results for the early detection of glaucoma using imaging such as fundus photographs, or optical coherence tomography.^3^ Two common applications include classifying glaucoma disease and segmenting the optic disc (OD). Researchers have used deep learning (DL) to either tackle these tasks separately^4^^–^^8^ or combine them by first localizing^9^^–^^11^ the OD^12^^–^^16^ and then using this region of interest to classify images. Forty seven recent studies on commonly used public fundus data sets have been summarized in terms of study count for both glaucoma classification and OD segmentation tasks (Table 1).^4^^–^^7^^,^^11^^,^^12^^,^^14^^,^^15^^,^^17^^–^^55^

**Table 1.** Summary of 50 Recent Deep Learning Studies^4^^–^^7^^,^^11^^,^^12^^,^^14^^,^^15^^,^^17^^–^^55^ for Glaucoma Diagnosis

	Study Count (Total Number = 50)	
Data Set	Glaucoma Classification	OD Segmentation
Public		
RIGA^64^	—	9
RIM-ONE^65^	15	14
Drishti-GS^66^	17	17
REFUGE^67^	12	18
RWD	8	1
RWD + public	2	1

The table lists the data sets and tasks used in each publication. Some publications use multiple data sets and perform multiple tasks.

Furthermore, many studies have validated DL models for glaucoma diagnosis using commonly used public data and have achieved promising results (Table 1). However, deploying these AI models in clinical settings is still a challenge.^56^^–^^58^ To successfully implement AI in the clinic, it is crucial to assess the generalizability of the models to real-world data (RWD), which can vary in parameters such as image acquisition protocols, imaging devices, ancillary hardware, patient populations, and health care settings.^59^ However, limited literature exists on evaluating the generalizability of DL models trained on commonly used public data to RWD (last row of Table 1). This lack of evaluation raises questions about the usefulness of publicly trained DL models for future use in the clinic to screen, detect, and prevent glaucoma.

Recently, studies on various nonophthalmic applications have demonstrated the significance of evaluating the generalizability of DL models for successful deployment in the clinic. For example, Mårtensson et al.,^60^ Rasmy et al.,^61^ and Li et al.^62^ have shown that DL models trained on homogeneous data can perform poorly when tested on heterogeneous clinical RWD in applications such as predicting medial temporal atrophy and heart failure, as well as handling epistasis. These studies evaluated the generalizability of DL models using diverse data sources such as magnetic resonance imaging (MRI), electronic health record, and single-nucleotide polymorphism data sets. However, they were not conducted on ophthalmic applications or ophthalmic data, and they did not use similar DL methods. Mojab et al.^63^ conducted a study on DL model performance and generalizability using manually selected pristine versus noisy fundus RWD for glaucoma detection. In this current study, we extend the data and AI tasks studied by Mojab et al.^63^ and provide a more comprehensive quantitative and qualitative analysis.

The goal of this study is to assess the generalizability of DL models trained on commonly used public fundus data sets to an instance of RWD for glaucoma diagnosis. Generalizability assessment will be performed on two commonly studied tasks for glaucoma diagnosis, glaucoma disease classification, and OD segmentation. For the glaucoma classification task, the results of the deep learning algorithms will be compared to the manual performance of glaucoma specialists, and the gradient-weighted class activation mapping (Grad-CAM) method will be used to aid in interpretation. Further, the susceptibility of DL models trained on public data versus RWD to the shift in data will be examined using t-distributed stochastic neighbor embedding (t-SNE).

We hypothesized that (1) the commonly used public data sets come from tight clusters, implying a lack of data diversity in contrast to RWD, and thus (2) the public-trained DL models have limited ability to generalize to RWD.

## Data

### Data Source

We used a subset of fundus images from the Department of Ophthalmology and Visual Sciences at the Illinois Eye and Ear Infirmary (IEEI) of the University of Illinois Chicago.^68^ IEEI data were anonymized, and the study was approved by an internal institutional review board and adhered to the tenets of the Declaration of Helsinki. We used billing information (International Classification of Diseases codes) from the University of Illinois Hospital and Health Sciences (UIH) as our binary labels for the glaucoma classification task. IEEI data are collected in a real-world setting, so we used this data set as an instance of RWD. IEEI fundus images are heterogeneous for the following reasons: (1) various imaging conditions such as different device use, operators (i.e., imaging personnel) with varying degrees of training, sensor quality, camera angle, or lighting conditions; (2) difference in dimension and resolution; (3) realism due to data collection in an uncontrolled or unposed setting, resulting in blurry, washed-out, or distorted images by motion artifacts; (4) racial and ethnic diversity; and (5) a large amount of missing data due to data collection over 12 years from multiple sources (e.g., hospital, imaging department, billing system).^68^

**Figure 1.**
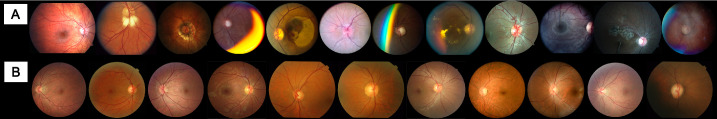
A random sample of fundus images from (**A**) IEEI (an instance of real-world data) and (**B**) commonly used publicly available data sets.

We utilized six commonly used public fundus data sets, including RIGA^69^ (comprising MESSIDOR, Bin Rushed, and Magrabi), Drishti-GS,^66^ REFUGE,^67^ and RIM-ONE DL.^65^ The RIGA data set included OD segmentation masks but lacked glaucoma classification labels. Therefore, we used RIGA images for OD segmentation and images from Drishti-GS, REFUGE, and RIM-ONE DL for glaucoma classification analysis. Figure 1 presents a random sample of fundus images, with parts (A) and (B) representing the IEEI and commonly used public images, respectively. We further discussed the data specifications between RWD and public data in terms of fundus camera, disease level, and image quality in Section 1 of the supplemental material.

### Training/Testing Data for Segmentation

We used a subset of the publicly available RIGA data set,^64^^,^^69^ which includes three sets of fundus images: MESSIDOR, Bin Rushed, and Magrabi data sets. Each set respectively comprises 460, 195, and 95 total images. Six glaucoma experts independently annotated the OD in each image of the RIGA data set.^64^^,^^69^ We collected manual OD annotations for 350 randomly selected images from our RWD sample by a third-year medical student. We thus selected 350 images randomly from the combined public data sets by choosing all 95 images from the Magrabi data set and a similar number of images (130 and 125) from the MESSIDOR and Bin Rushed data sets. We then treated the combined public data sets as one data set and split the data into three sets with approximate ratios of 56% (*n* = 200) for training, 14% (*n* = 50) for validation, and 30% (*n* = 100) for testing. We split RWD with the same approximate ratios into the train, validation, and test sets. Patient information for the RIGA data set is not available. We split the RWD by patient to prevent the leakage of information from the test set into the training. Table 2 shows the breakdown of train, validation, and test sets randomly selected per RWD and public data.

**Table 2.** Selected Public Data and RWD for the OD Segmentation Task

	Number of Images (Number of Participants)			
Data Set	RWD	MESSIDOR	Bin Rushed	Magrabi
Train	200 (88)	70	70	55
Validation	50 (27)	20	20	10
Test	100 (39)	40	35	30
Total	350 (154)	350		

The number of participants is specified in parentheses for data sets where that information is available.

### Training/Testing Data for Classification

We used subsets of three other commonly used publicly available fundus data sets for the glaucoma classification task since the RIGA data set does not have glaucoma classification labels. We used the publicly available Drishti-GS^66^ (*n* = 101), RIM-ONE DL^65^ (*n* = 485), and REFUGE^67^ (*n* = 1,200) data sets along with our RWD. We used all 361 available glaucomatous images in the combined three public data sets and randomly sampled 361 nonglaucoma images from the remaining images in the Drishti-GS, RIM-ONE DL, and REFUGE data sets. For the RWD, we randomly chose 361 images per class. We treated the combined public data sets as one data set (*n* = 722) and split the data into three sets with approximate ratios of 70% (*n* = 520) for training, 10% (*n* = 70) for validation, and 20% (*n* = 130) for testing. We split the RWD (*n* = 722) with the same approximate ratios into the train, validation, and test sets. While patient information for the RIM-ONE DL and REFUGE data sets is unavailable, we split the RWD by patient and used the entire Drishti-GS data in the train set. Table 3 shows the breakdown of train, validation, and test sets randomly selected per RWD and public data.

**Table 3.** Selected Public Data and RWD for the Glaucoma Classification Task

	Number of Images (Number of Participants)			
Data Set	RWD	Drishti-GS	RIM-ONE-DL	REFUGE
Train	520 (392)	101 (68)	237	183
Validation	62 (51)	0	45	26
Test	140 (97)	0	90	40
Total	722 (540)	722		

The number of participants is specified in parentheses for data sets where that information is available.

### Manual Labels for Glaucoma Classification by Experts

We compared the performance of our DL glaucoma classifiers with two glaucoma specialists by collecting labels for test images in the RWD and public data sets. The specialists assigned each image to one of five classes: nongradable, anomalous, nonglaucoma, probable glaucoma, and definite glaucoma. Images were assigned to the nongradable class if they had insufficient signals for physicians to diagnose glaucoma, caused by excessive noise such as opacity, brightness, darkness, motion artifacts, blur, or washed-out effects. Images were assigned to the anomalous class if they showed anomalous features in the optic disc area, such as abnormal size, shape, color, or vasculature. Physicians assigned images to probable and definite glaucoma classes based on characteristics of the optic disc. Definite glaucoma class was assigned if the following glaucomatous optic nerve characteristics were detected: focal or generalized thinning of the neuroretinal rim less than 0.1, the characteristic wedge-shaped retinal nerve fiber layer defect, or disc hemorrhage. Probable glaucoma was assigned if the enlarged cup-to-disc ratio greater than or equal to 0.6 was detected without the presence of focal or generalized thinning of the neuroretinal rim, the retinal nerve fiber defects, or disc hemorrhage.

Table 4 shows the breakdown of physicians’ manual labeling on test images in RWD and public data sets. We combined definite and probable glaucoma in our analysis and excluded nongradable and anomalous images based on recommendations from experts.

**Table 4.** Statistics on Experts’ Manual Labeling of RWD and Public Data

	Number of Images Per Class			
Test Set	Definite/Probable Glaucoma	Nonglaucoma	Nongradable	Anomalous
RWD	63	67	3	7
Public	68	52	8	2

## Methods

### Data Visualization Via t-SNE

We employed the ResNet-50^70^ model to extract features from the RWD and public data and t-SNE method^71^ to visualize those feature maps. Specifically, first, we employed a pretrained ResNet-50 model based on PyTorch implementation to extract 2048 feature maps from the last convolutional layer of the ResNet-50 backbone. Second, we used t-SNE for dimensionality reduction and visualization of 2048 feature maps into a two-dimensional feature space. We measured the spread of t-SNE results by calculating the trace of the covariance matrix for each data set (i.e., RWD, MESSIDOR, Bin Rushed, Magrabi, Drishti-GS, and REFUGE). We analyzed RIM-ONE DL images separately from the rest of the full-view fundus data sets since they were originally cropped. We analyzed RIM-ONE DL t-SNE results with the other two data sets (i.e., Drishti-GS and REFUGE) we cropped to use in the glaucoma classification task.

### Development of the Deep Learning Algorithm

#### Generating Pseudo-Labels

RIM-ONE-DL has the largest glaucoma sample among the chosen public data sets. Fundus images in the RIM-ONE-DL data set are originally cropped around the OD region. We similarly cropped all other images used in our glaucoma classification experiments. To automatically crop a fundus image, we used the corresponding OD segmentation mask of that image. While all images in the public data have OD segmentation masks, only 350 (out of 722) images in RWD have OD masks. Therefore, as indicated in Figure 3A, we first trained an OD segmentation model using the 350 labeled subset of images and then used that model to infer OD masks for the 372 unlabeled images in RWD, called pseudo-labels. We generated pseudo-labels using our best-performing OD segmentation model.

#### Cropping Fundus Images

By generating pseudo-labels for the unlabeled sample of RWD, all images used in our glaucoma classification experiments have OD segmentation masks. Therefore, as indicated in Figure 3C, we cropped images using their OD masks. This procedure includes finding the contours of OD masks, calculating the center and radius of OD masks, laying OD masks over the original retinal images, and then cropping the OD region by a bounding box with the mask's center and its radius plus an arbitrary margin (i.e., 40 pixels).

#### Experimental Setup and Trained Networks

**Figure 2.**
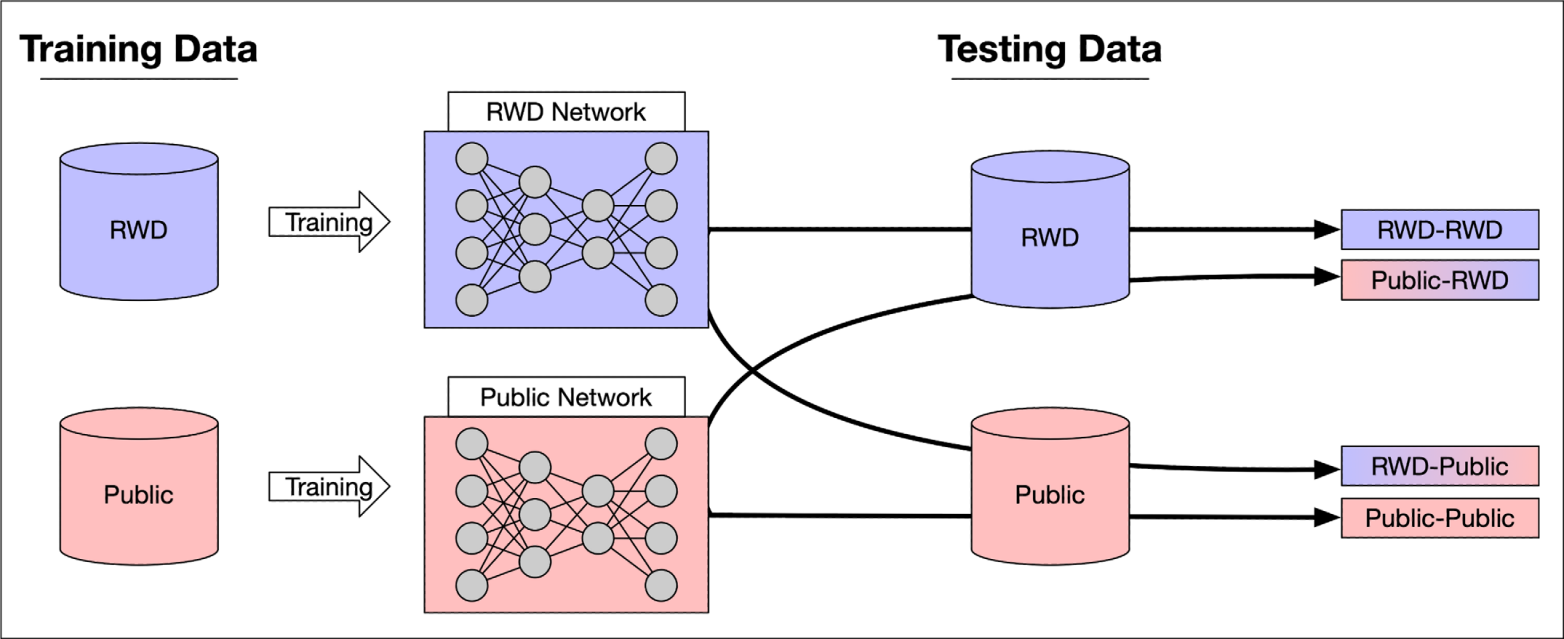
Schematic view of experimental setup for OD segmentation and glaucoma classification tasks.

We created two models per task of OD segmentation and glaucoma classification. Per task, one model was trained on public data, and the other model was trained on the RWD. To investigate the generalizability of the two trained models, we tested each model, per task, on all different data sets (i.e., RWD and public). We will refer to each model as a train–test pair (e.g., the model trained on RWD and tested on public data is the RWD–Public model). Figure 2 indicates a schematic view of our experimental setup for training and testing models for each task. We discussed the evaluation metrics for each task in Section 2 of the supplemental material.

##### Implementation Details for OD Segmentation

**Figure 3.**
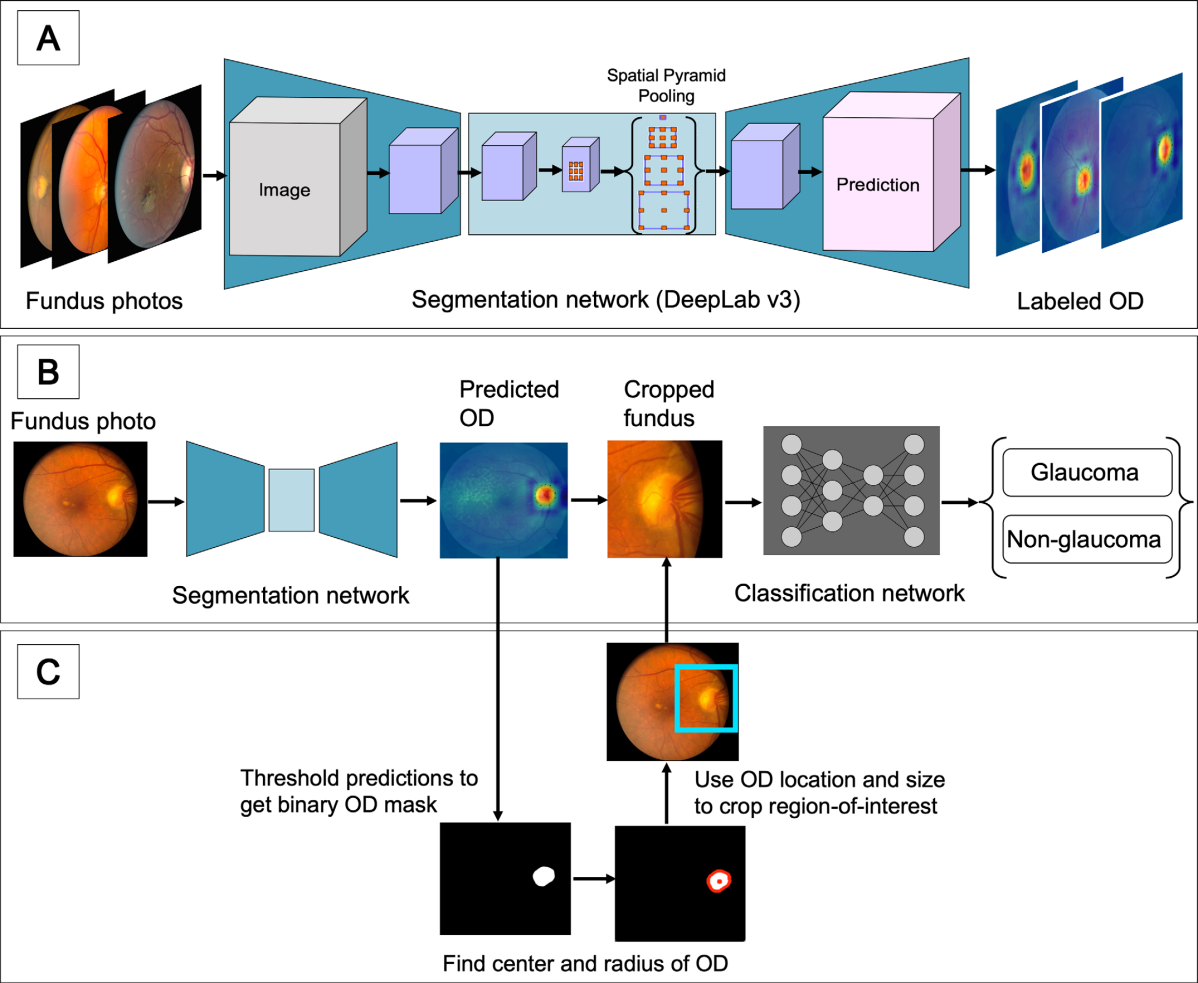
Pipeline flowchart. (**A**) OD segmentation pipeline. (**B**) Glaucoma classification pipeline. (**C**) Fundus image cropping pipeline.

We trained two OD segmentation models using the DeepLabv3^72^ network with the ResNet-50^70^ backbone on RWD and public data, as indicated in Figure 3A. The DeepLabv3-ResNet50 model was based on PyTorch implementation, pretrained on a subset of COCO train 2017. We set the model’s output to 1 and optimized the binary cross-entropy loss function with the ADAM gradient descent optimizer. Input fundus images were resized to 256 × 256 pixels. We performed six hyperparameter (HP) searches for each of the two data sets (RWD and public) and selected the best model based on the maximum validation accuracy over HP searches, per data set. The hyperparameters included learning rate ({10^−2^, 10^−3^, 10^−4^}), batch size ({128, 64, 32, 16}), weight decay for *l*_2_ regularization ({10^−5^, 10^−4^, 0}), and data augmentation techniques ({random horizontal flip, random rotation, random translation}). Rotation angles were randomly chosen from {0°,  90°,  180°,  270°}, and translation was randomly performed by shifting the image up to 50 pixels in both *x* and *y* directions. Additional hyperparameters consisted of image color conversion choices (RGB, grayscale) and binary choices on whether to normalize each image using its mean and standard deviation. We trained the networks for 2000 epochs.

##### Implementation Details for Glaucoma Classification

We trained two glaucoma classification models using the ResNet-50^70^ architecture on RWD and public data. The ResNet-50 model was based on PyTorch implementation, pretrained on the ImageNet^73^ data set. We set the model’s output to 2 and optimized the cross-entropy loss function with the ADAM gradient descent optimizer. As indicated in Figure 3B, cropped fundus images were used as inputs to the model, and they were resized to 128 × 128 pixels. We performed 20 HP searches for each of the two data sets (RWD and public) and selected the best model based on the maximum validation accuracy over HP searches, per data set. The hyperparameters included learning rate ({10^−2^, 10^−3^, 10^−4^}), batch size ({128, 64, 32, 16}), weight decay for *l*_2_ regularization ({10^−5^, 10^−4^, 0}), and data augmentation techniques ({random horizontal flip, random rotation, random translation}). Rotation angle was randomly chosen from {0°,  90°,  180°,  270°}, and translation was randomly performed by shifting the image up to 12 pixels in both *x* and *y* directions. Additional hyperparameters consisted of image color conversion choices (RGB, grayscale) and binary choices on whether to apply contrast limited adaptive histogram equalization (CLAHE). Early stopping was used on the validation. The training process was initiated with 3000 epochs and terminated if the validation accuracy did not improve for 200 consecutive epochs.

### Qualitative Assessment of Glaucoma Classification

#### Visualizing Model Understanding

We applied the Grad-CAM^74^ visualization method to explore the knowledge learned by RWD and public glaucoma classification models and understand their decision-making process on glaucoma. We evaluated the Grad-CAM results on the test sets of both public data and the RWD using both public-trained and RWD-trained models.

#### Models’ Feature Map Visualization via t-SNE

We used the RWD model and the public model to extract 2048 feature maps from the last convolutional layer of their backbones. We visualized these feature maps in a two-dimensional space using the t-SNE^71^ method. We measured the similarity between the t-SNE results from the public model and the RWD model using the Wasserstein distance.^75^

## Results

### Data Visualizations Via t-SNE

**Figure 4.**
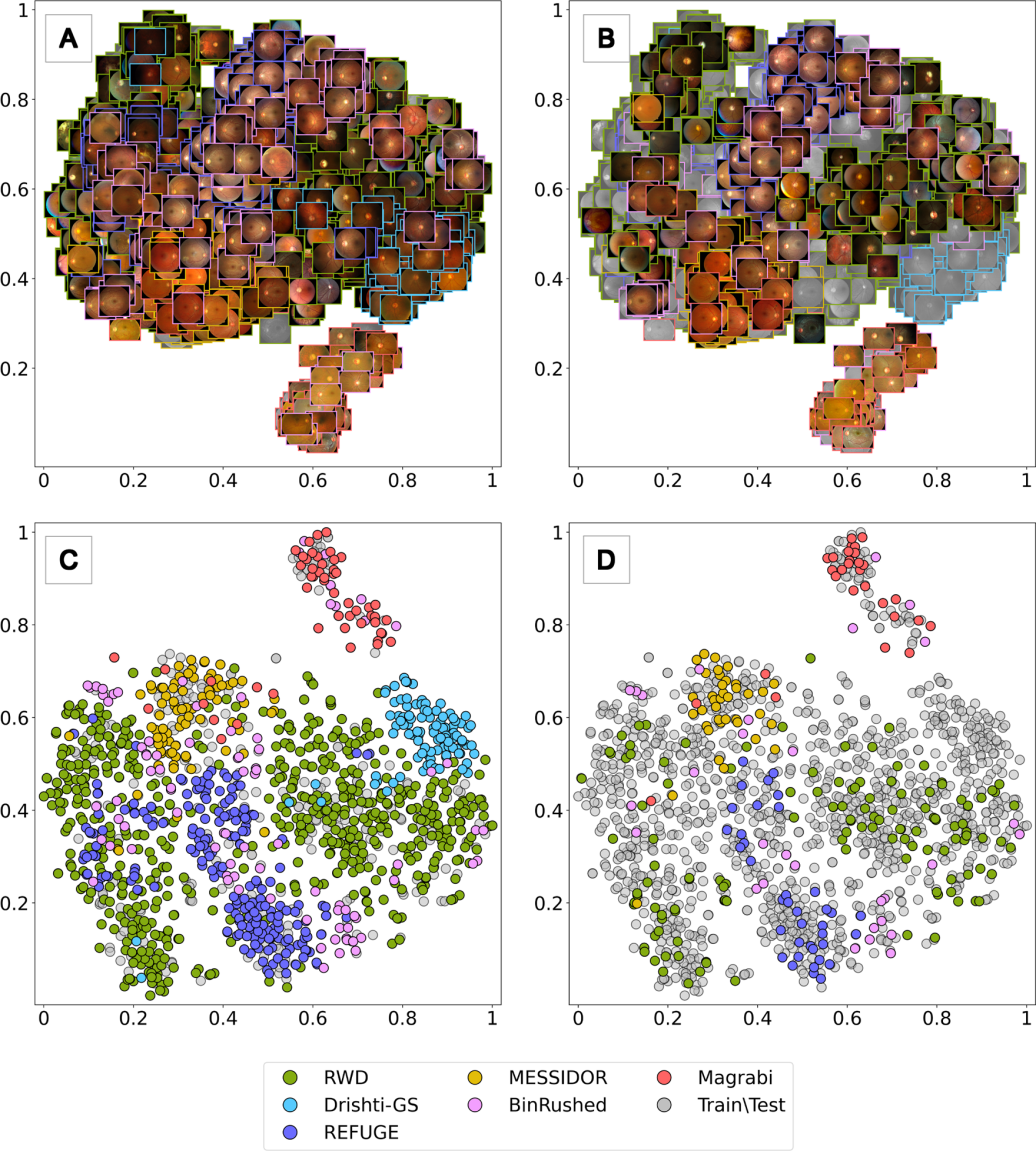
ResNet-50 features visualization for most of the commonly studied public fundus data sets and an instance of RWD used in this study via t-SNE. t-SNE results shown as original input images for (**A**) train sets and (**B**) test sets. t-SNE results shown as scatter points for (**C**) train sets and (**D**) test sets. In parts (**A**) and (**C**), the gray color represents test data on the train set plots. Conversely, in parts (**B**) and (**D**), the gray color represents train data on the test plots.

Figure 4 presents a t-SNE visualization of feature maps for the entire sample of RWD and the public data used in this study. However, the RIM-ONE-DL images, due to their cropped nature, are not included in Figure 4. The t-SNE results for the cropped images used in the classification analysis can be found in Section 3 of the supplemental materials. Figure 4 showcases the t-SNE results for the other data sets, each represented by a unique color. Parts (A) and (C) display the t-SNE results for the training data sets, while parts (B) and (D) show the t-SNE results for the test data sets. In parts (A) and (B), the t-SNE results are visualized using a full-view representation of the fundus images, while parts (C) and (D) present the same t-SNE results as scatterplots. In parts (A) and (C), the gray color represents test data on the train set plots. Conversely, in parts (B) and (D), the gray color represents train data on the test plots. This color scheme aids in distinguishing between the training and test data sets in the t-SNE visualizations.

The Bin Rushed data set exhibited the largest spread in its t-SNE projections, with a covariance matrix trace of 0.12. The other data sets ranked by decreasing trace values were RWD, REFUGE, Magrabi, Drishti-GS, and MESSIDOR, with covariance traces of 0.11, 0.04, 0.03, 0.02, and 0.01 respectively, indicating lower spread in their t-SNE projections. Table 5 shows the feature spread (TSNE covariance trace) and similarity (Wasserstein distance) for the union of public data sets and RWD shown in Figure 4. The trace of the t-SNE covariance matrix for the union of public train and test sets is 0.108 and 0.110, respectively. For the RWD train and test sets, the values are 0.108 and 0.104. The Wasserstein distances for both the public train and test sets and the RWD train and test sets are 0.003. The Wasserstein distance is 15.8 between the public and RWD train sets and 27.1 between their test sets.

**Table 5.** Comparison of Feature Spread (t-SNE Covariance Trace) and Similarity (Wasserstein Distance) between the Union of Public Data Sets and RWD Using Off-the-Shelf ResNet-50 Model

	Between Data Sets				Within Data Sets			
Metrics	Public_tr_	RWD_tr_	Public_te_	RWD_te_	Public_tr_	Public_te_	RWD_tr_	RWD_te_
Feature spread (covariance trace)	0.108	0.108	0.110	0.104	0.108	0.110	0.108	0.104
Feature similarity (Wasserstein distance)	15.8		27.1		0.003		0.003	

*te*, test set; *tr*, train set.

### Optic Disc Segmentation

We summarized the comparison results of public versus RWD models for the OD segmentation task in Table 6. Each row of the table shows the performance of the best-selected model for each train–test pair. We found that the model trained on public data and tested on public data (i.e., public–public) segments OD with a mean intersection over union (IoU) of 93.6%. The public–public and RWD–public models have 95% confidence intervals (CIs) of 93.5%–94.0% and 90.8%–92.1% around their mean IoU, respectively. Consequently, the difference between the mean IoU of the public–public and RWD–public models is 2.5%. The difference between the mean IoU of the public–public model and the public–RWD model is 8.0%. Further, the (RWD + public) model attained a mean IoU of 94.0% on the public test set, with a 95% CI of 93.4%–94.5%. Therefore, we observed a difference of 0.4% between the mean IoU of the public–public model with that of the (RWD + public)–public model.

**Table 6.** Results of Public Model versus RWD Model for the OD Segmentation Task

Data Set		Evaluation Metrics on Test Set (95% CI), %		
Train	Test	Mean IoU	Mean SEN	Mean PPV
RWD	RWD	87.6 (87.0–88.8)	93.4 (93.0–94.7)	93.2 (92.8–93.9)
Public		85.6 (84.9–86.7)	93.2 (92.7–94.6)	91.8 (91.4–92.6)
(RWD + Public)		88.2 (87.3–88.9)	95.0 (93.6–95.3)	94.0 (93.0–94.0)
Public	Public	93.6 (93.5–94.0)	97.3 (97.2–97.5)	96.2 (96.0–96.6)
RWD		91.1 (90.8–92.1)	94.5 (94.2–95.5)	96.4 (96.2–96.9)
(RWD + Public)		94.0 (93.4–94.5)	97.5 (95.8–96.9)	97.1 (97.4–97.7)

PPV, positive predicted value; SEN, sensitivity.

We also found that the RWD–RWD model segments OD with a mean IoU of 87.6%. The RWD–RWD and public–RWD models have a 95% CI of 87.0%–88.8% and 84.9%–86.7% around their mean IoU, respectively. Therefore, the difference between the mean IoU of the RWD–RWD and public–RWD models is 2.0%. Further, the (RWD + public) model attained a mean IoU of 88.2% on the RWD test set, with a 95% CI of 87.3%–88.9%. Therefore, we observed a difference of 0.6% between the mean IoU of the RWD–RWD model with that of the (RWD + public)–RWD model. Table 6 shows similar results for other metrics, including sensitivity and precision, across models and data sets. The comparison of the RWD model and public model performance on each image in the public and RWD test sets can be found in Section 4 of the supplemental materials.

### Glaucoma Classification

We summarized the comparison results of public versus RWD models for the glaucoma classification task in Table 7. Each row of the table shows the performance of the best-selected model for each train–test pair. We found that the model trained on public data and tested on public data (i.e., public–public) classifies glaucoma with an accuracy of 87.6% and mean area under the receiver operating characteristic curve (AUROC) of 95.0%. The public–public and RWD–public models have a 95% CI of 94.3%–96.5% and 84.5%–88.5% around their mean AUROC, respectively. Therefore, the difference between the accuracy and mean AUROC of the public–public model and RWD–public model is 6.8% and 8.9%, respectively. Further, the difference between the accuracy and mean AUROC of the public–public model and the public–RWD model is 15.5% and 18.4%, respectively. Further, the (RWD + public) model attained an accuracy of 84.1% and mean AUROC of 92.6% on the public test set, with a 95% CI of 91.7%–94.7% around AUROC. Therefore, we observed the difference between the accuracy and mean AUROC of the public–public model and (RWD + public)–public model is 3.5% and 2.4%, respectively.

**Table 7.** Results of Public versus RWD Models for the Glaucoma Classification Task

	Data Set		Evaluation Metrics on Test Set (%)				
Predictor	Train	Test	Accuracy	SEN	PPV	*F*_1_	AUROC (95% CI)
ResNet-50	RWD	RWD	80.6	76.9	75.4	76.1	86.1 (84.7–88.1)
ResNet-50	Public		72.1	50.8	72.5	59.7	76.6 (74.5–79.0)
ResNet-50	RWD + Public		80.0	80.7	72.4	76.3	85.2 (83.7–87.5)
Physician	—		77.6	88.7	71.4	79.1	—
ResNet-50	Public	Public	87.6	90.6	76.4	82.9	95.0 (94.3–96.5)
ResNet-50	RWD		80.8	74.4	68.0	71.1	86.1 (84.5–88.5)
ResNet-50	RWD + Public		84.1	88.3	73.0	80.0	92.6 (91.7–94.7)
Physician	—		79.1	88.2	77.9	82.7	—

We also found that the RWD–RWD model classifies glaucoma with an accuracy of 80.6% and mean AUROC of 86.1%. The RWD–RWD and public–RWD models have a 95% CI of 84.7%–88.1% and 74.5%–79.0% around their mean AUROC, respectively. Therefore, the difference between the accuracy and mean AUROC of the RWD–RWD and public–RWD models is 8.5% and 9.5%, respectively. Further, the (RWD + public) model attained an accuracy of 80.0% and mean AUROC of 85.2% on the RWD test set, with a 95% CI of 83.7%–87.5% around AUROC. Therefore, we observed the difference between the accuracy and mean AUROC of the RWD–RWD model and (RWD + public)–public model is 0.6% and 0.9%, respectively. Table 3 shows similar results for other metrics, including sensitivity, precision, and *F*_1_ score, across models and data sets.

**Figure 5.**
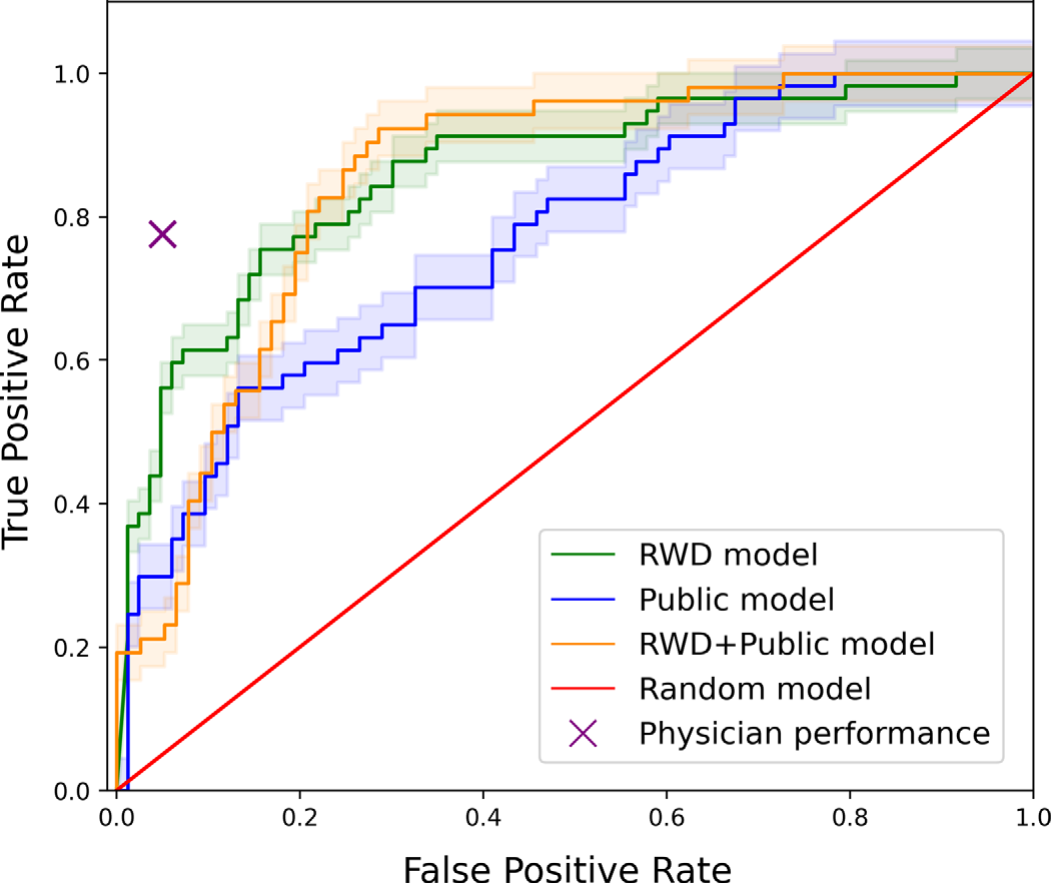
Comparison of RWD model's, public model's, and RWD + public model's AUROC curves with physicians’ accuracy for glaucoma classification on the RWD test set.

Figure 5 shows a comparison of the performance of the RWD model, public model, (RWD + public) model, and physicians for classifying glaucoma on the RWD test set. The AUROC curves for the RWD, public, and (RWD + public) models are shown in green, blue, and orange, respectively, and the accuracy of physicians is represented by a purple cross. The *x* coordinate for physicians’ accuracy is arbitrary. We further showed the AUROC plots for RWD–RWD, public–public, RWD–public, public–RWD, (RWD + public)–RWD, and (RWD + public)–public models in Section 5 of the supplemental materials. In addition, we showed the results of using the RWD model versus public model on a larger (*n* = 5275) unseen sample of the RWD in Section 5.1 of the supplemental materials.

Table 7 also shows the physicians’ classification performance using images in RWD and public test sets. The accuracy of the physicians, compared to ground-truth labels, in classifying glaucoma was 77.6% in the RWD test set and 79.1% in the public test set. The accuracy of the glaucoma classifier trained on RWD was 1.7% more compared to physician performance in the public test set and 3.0% more compared to physician performance in the RWD test set. The accuracy of the public classifier was 8.5% more compared to physician performance in the public test set and 5.5% less compared to physician performance in the RWD test set. Further, the accuracy of the RWD + public classifier was 5.0% more compared to physician performance in the public test set and 2.4% less compared to physician performance in the RWD test set.

### Assessment of Glaucoma Classifiers

#### Model Decision-Making Visualization

**Figure 6.**
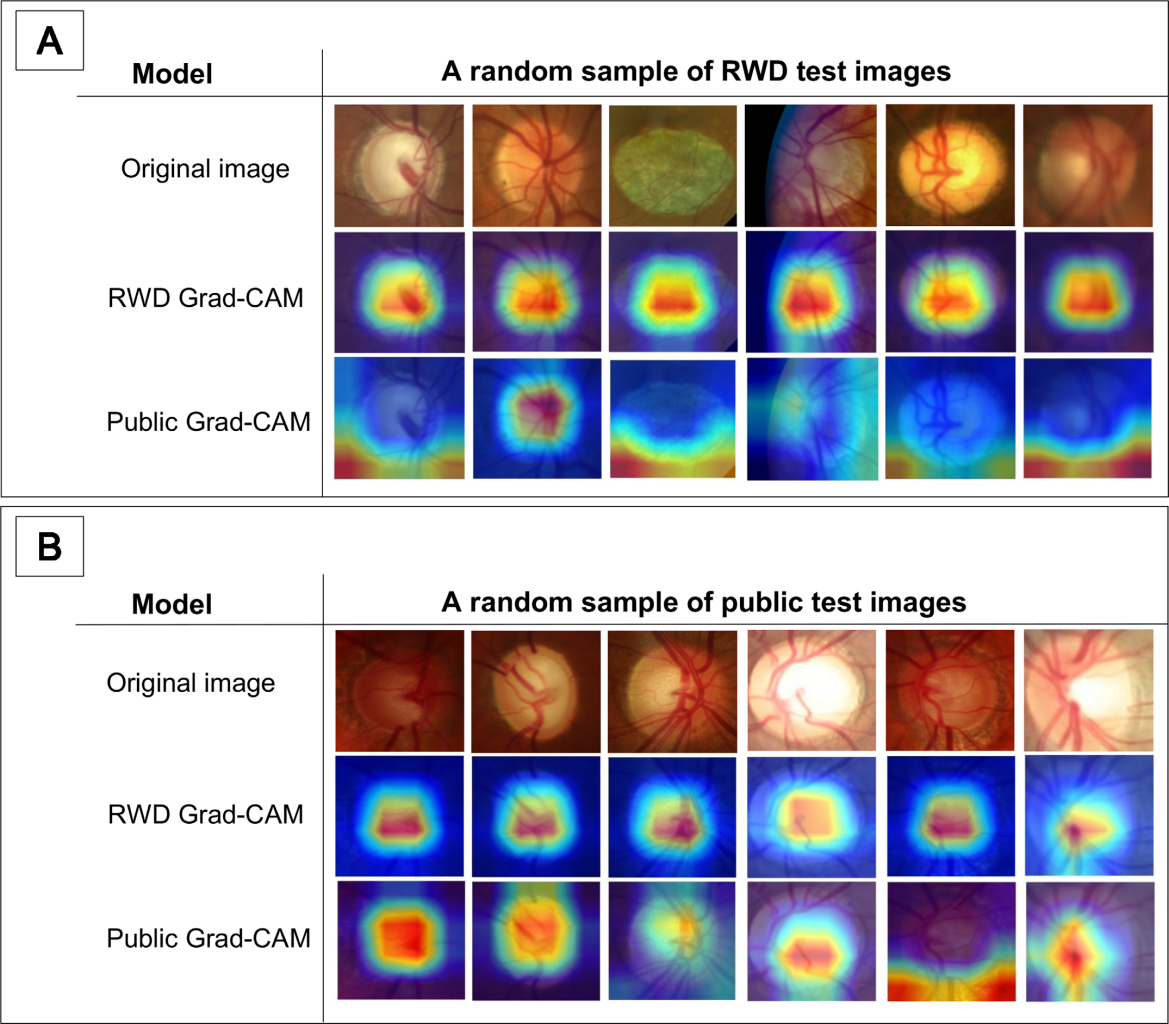
Grad-CAM visualization for the glaucoma classification task. Grad-CAM predictions on a handful number of randomly sampled (**A**) RWD and (**B**) public test images.

Figure 6 shows the results of Grad-CAM prediction for a random sample of test images from RWD and public data. The original images are displayed in the top row, and Grad-CAM predictions for the last convolutional layer in the last residual block of the RWD and public models are displayed in the middle and last rows, respectively. The red part of the Grad-CAM heatmap represents the chosen class. Table 8 shows the quantitative Grad-CAM prediction results using the RWD-trained and public-trained models. The RWD-trained model predicts glaucoma based on features inside the OD region in fundus images for 94.2% and 96.9% of the test images in RWD and public data, respectively. The public-trained model predicts glaucoma based on features inside the OD region for 67.1% and 71.5% of the test images in RWD and public data, respectively. The public-trained model predicts glaucoma based on features further away from the OD region for a higher percentage of images (27.1% and 25.4%) in the RWD and public test sets compared to the RWD-trained model.

**Table 8.** Grad-CAM Results for the Glaucoma Classification Task

Data Set		Grad-CAM Prediction Region in Fundus Image	
Train	Test	Number (%) Inside of OD	Number (%) Outside of OD
Public	RWD	94 (67.1)	46 (32.8)
RWD		132 (94.2)	8 (5.7)
Public	Public	93 (71.5)	37 (28.4)
RWD		126 (96.9)	4 (3.0)

#### Models’ Feature Map Visualization Via t-SNE

**Figure 7.**
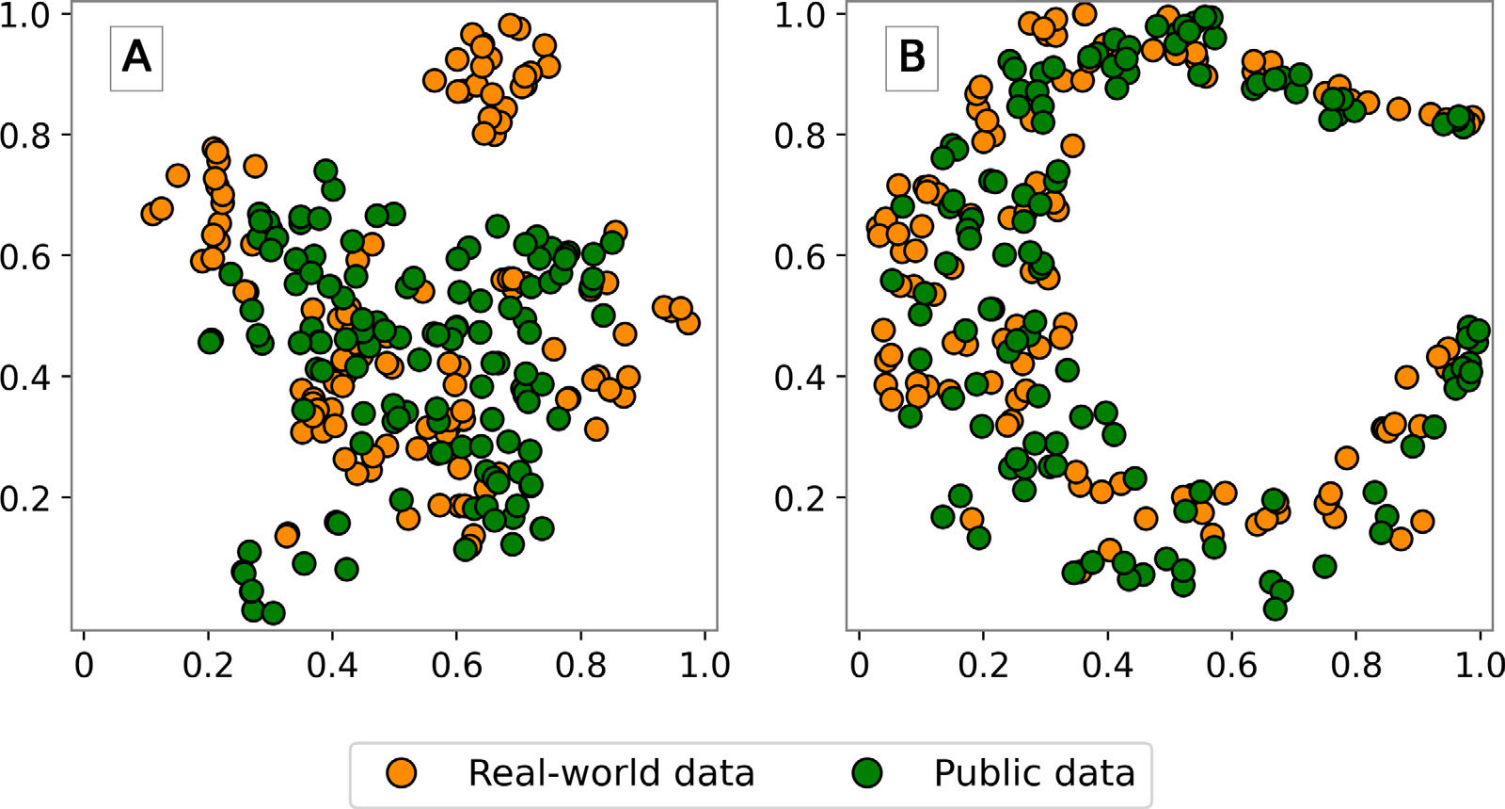
t-SNE visualization of feature maps learned by (**A**) public model and (**B**) RWD model on all test data sets (i.e., public data, RWD).

Figures 7A and 7B display the two-dimensional projections of feature maps learned by the public and RWD models, respectively, for all test data sets, which include both RWD and public data. In Figure 7A, the Wasserstein distance between the feature maps of the RWD and public test sets, computed using the public model, is 0.140. In Figure 7B, this distance using the RWD model is 0.060. We elaborated on the Wasserstein distances and spread of features per model and data set in Table 3 of Section 3 of the supplementary materials. Also, we separately showed feature representations for public–public, RWD–RWD, public–RWD, and RWD–public experiments in Figure 4 of Section 5.2 of the supplementary materials.

## Discussion

Evaluating the performance of deep learning models on real-world data is crucial for successful deployment in clinical settings. We compared the performance of DL models trained on commonly used public data to an instance of RWD for two common AI tasks in glaucoma diagnosis: disease classification and OD segmentation. Our main findings (1) established the importance of using RWD for developing generalizable DL models in glaucoma diagnosis, particularly for the classification task; (2) supported that the union of the public data exhibits a comparable level of diversity to the RWD while their feature distributions are distinct; and (3) demonstrated the RWD-trained glaucoma classifier more closely resembles the decision-making process of glaucoma physicians when compared to public data-trained models.

Specifically, to evaluate the diversity of data sets, we used the t-SNE method to visualize ResNet-50 features extracted from RWD and public data sets in two dimensions to identify relevant patterns in the data, measuring spread by two-dimensional variance. Our results showed that except for Bin Rushed, the RWD had a greater than 175% spread in the t-SNE projection compared to commonly used public data sets like Drishti-GS,^66^ REFUGE,^67^ MESSIDOR,^69^ and Magrabi.^69^ However, when considering the union of the public data sets versus RWD, we found a relatively similar spread of features. This was supported by the comparable trace of the covariance associated with their respective features, as depicted in Figure 4, Table 5, and Supplementary Figure S1, Supplementary Table S2, and Supplementary Table S3. Although the trace of the covariance of t-SNE features extracted by the off-the-shelf ResNet-50 model, RWD model, or public model indicates similar levels of variance between the RWD and public data sets, drawing conclusive insights regarding the heterogeneity within each data set is challenging. Nonetheless, the relatively large Wasserstein distance between the two data sets suggests a notable difference in their distributions, implying that the RWD and public data sets may be out-of-distribution with each other. This finding potentially contributes to explaining the encountered difficulties in achieving generalizability between models trained on each respective data set.

The performance of DL models in terms of generalizability differs for the two tasks, the OD segmentation and glaucoma classification. Specifically, for OD segmentation, we found that models trained on either RWD or public data performed similarly on unseen RWD. Although the model trained on RWD statistically significantly outperformed the public model by 2%, the difference may not be clinically significant. Thus, the use of public-trained data could be seen as effective as the use of RWD-trained data for fundus image OD segmentation. Additionally, we observed a less than 1% improvement in mean IoU for a model trained using a combined data set of RWD and public compared to models trained on either data set alone. These findings suggest that the model trained on the combined data set performs comparably to the models trained on either data set for the OD segmentation task.

In contrast, for glaucoma classification, the model trained on public data showed limited generalization to RWD, while the model trained on RWD outperformed the public model by 8.5% in terms of accuracy and 9.5% in terms of mean AUROC on RWD. This suggests that relying solely on public data may be insufficient for developing generalizable DL models for glaucoma classification and that incorporating RWD is necessary to improve the generalizability of DL models. Additionally, despite the larger training set size obtained by combining the RWD and public data sets, we did not observe an improvement in accuracy for either the RWD or public test sets.

Additionally, the differing generalization results between segmentation and classification using public data versus RWD could be attributed to multiple factors. First, the inherent generalizability of the tasks themselves: studies by Mojab et al.^76^ have demonstrated that segmentation tasks are typically more generalizable than classification tasks due to their robustness against overfitting. Srivastava et al.^77^ have shown that classification networks tend to overfit, hindering their generalizability. Second, the relative difficulty of the tasks may also play a role. OD segmentation is less complex, as the optic disc has distinct morphologic characteristics such as a bright region and circular shape that are easier for deep learning networks to identify. In contrast, glaucoma classification involves more subtle and complex biomarkers, such as cup-to-disc ratio, thinning of the neuroretinal rim area, or disc hemorrhages, which can be more challenging for a DL network to learn.

We demonstrated that the RWD glaucoma classifier is more accurate and closer in decision-making to glaucoma physicians compared to the public classifier. The RWD model’s accuracy differed from physicians by only 3.0% at most, compared to 8.5% for the public model. Our findings also showed that the RWD model is better aligned with domain knowledge on glaucoma, as it predicts 94.2% to 96.9% of glaucoma cases in test images based on features within the OD region, where most glaucoma biomarkers are located. In contrast, the public model predicts 28.4% to 32.8% of cases based on features further away from the OD region, which might not correlate with glaucoma key features. Thus, our results indicate that the RWD model not only is more generalizable but also predicts glaucoma more accurately compared to the public model.

While our study suggests RWD improve DL models’ generalizability in glaucoma classification, it has limitations. Publicly available RWD fundus image data sets for ophthalmic research are lacking. Our RWD sample is from a single institution, so the generalizability of DL models trained on public data to multi-institutional RWD is unknown. Further analysis is needed to evaluate the generalizability of DL models to multi-institutional RWD for glaucoma diagnosis and to make such data publicly available. Additionally, while our experts evaluated the image quality in RWD and public data, which is crucial for future analysis to determine the clinical usability of these images, the image quality was not taken into consideration in our DL models. To this end, we plan to develop DL models that can screen for poor-quality images (e.g., anomalous disc, blurry images) in our future study. Also, further analysis is needed to understand the limited improvement in OD segmentation and the lack of accuracy enhancement in glaucoma classification with a combined data set (i.e., RWD + public), warranting exploration of alternative data set combination methods and identification of influential factors.

## Conclusions

Recently, many studies in ophthalmology have used DL for diagnosing glaucoma on publicly available data sets. However, most of these studies have overlooked the evaluation of the generalizability of their proposed models to RWD. We showed the importance of RWD for a potential clinical translation of AI in glaucoma diagnosis. We demonstrated RWD, comprising a greater sample diversity, is required to create generalizable models, and commonly used public data sets might not be sufficient for this purpose. Our study showed that DL-trained models on public data have limited ability to generalize to RWD for glaucoma classification while they obtain comparable performances for OD segmentation. We also demonstrated that RWD-trained models obtain closer performance to glaucoma physicians for the glaucoma classification task. We hope that the results of this study will motivate a broad community of investigators, especially in ophthalmology research, to reconsider their evaluation of DL models before certifying these models for deployment.

## Supplementary Material

Supplement 1
