# Supplementary material for: Validating the Generalizability of Ophthalmic Artificial Intelligence Models on Real-World Clinical Data
Source: Transl Vis Sci Technol. 2023 Nov 3;12(11):8. doi: 10.1167/tvst.12.11.8 (PMC10629532; doi:10.1167/tvst.12.11.8)
Supplement: Supplement 1 [file tvst-12-11-8_s001.pdf]

1 Data Specifications

The data specifications for the commonly studied publicly available fundus datasets, including RIGA, Drishti-GS, REFUGE, RIM-ONE-DL, as well as the instance of RWD sample used in this study are summarized in Table 1. Also, as discussed in the “Manual Labels for Glaucoma Classification by Experts" section of the manuscript, two glaucoma physicians have manually labeled images in the test sets of RWD and public datasets. Some images from REFUGE, RIM-ONE DL, and RWD samples were labeled as non-gradable or anonymous by the physicians, and this information has been included in the "Image quality" column as "gradable/non-gradable". However, since the physicians did not label the data from Drishti-GS and RIGA datasets, the "Image quality" column for those two datasets has been filled with "details unavailable".

2 Evaluation Metrics

We evaluated the segmentation results with the Intersection over Union (IoU) which measures the area of overlap between the predicted OD mask and the ground truth OD mask divided by their union area as defined in Equation (1). Further, we evaluated the prediction results for glaucoma classification using accuracy (Acc), sensitivity (SEN), precision (PPV), F_1-score, as defined in Equations (2)-(5). Equation (1) shows the metrics used for evaluating the performance of our trained segmentation model. Where true positive (TP) indicates the number of pixels in the coverage areas between the ground truth mask and the predicted mask; False Positive (FP) expresses the number of pixels in the area where the pixel is classified only in the predicted area and is excluded belonging to the ground truth; False Negative (FN) is the number of pixels in the area where a pixel is classified only in the ground truth mask and is excluded belonging to the predicted mask area. Further, Equations (2)-(5) show the metrics used for evaluating the performance of our trained glaucoma classification model. Where TP (True Positive) represents the positive cases (i.e., glaucoma) predicted to be positive, FN (False Negative) represents the positive cases predicted to be negative (i.e., non-glaucoma), TN (True Negative) represents the negative cases predicted to be negative, and FP (False Positive) represents the negative cases predicted to be positive. We further evaluated Area Under the Receiver Operating Characteristic Curve (AUROC) which shows the trade-off between SEN (or true positive rate) and false positive rate (FPR), defined in equation (6), across different decision thresholds (e.g., 0.1, 0.5).

$$IoU = \frac{TP}{TP + FP + FN} \tag{1}$$

$$Acc = \frac{TP + TN}{TP + TN + FP + FN} \tag{2}$$

$$Sen = \frac{TP}{TP + FN} \tag{3}$$

$$PPV = \frac{TP}{TP + FP} \tag{4}$$

$$F_1 = 2 \times \frac{PPV \times Sen}{PPV + Sen} = \frac{2TP}{2TP + FP + FN} \tag{5}$$

$$FPR = \frac{FP}{FP + TN} \tag{6}$$

Table 1. Data specifications for the commonly used publicly available data and the instance of real-world data (RWD) used in this study.

**Dataset**		**Disease level**	**Acquisition device**	**Data source**	**Image quality**
**Public**	RIGA (Almazroa et al, 2018)	**Details unavailable**	(1) MESSIDOR; Topcon TRC NW6 non-mydriatic retinograph, (FoV) 45 degrees<br>(2) Bin Rushed: CanonCR2 non-mydriatic digital retinal camera; (FoV) 45 degrees<br>(3) Magrabi: Topcon TRC 50D Xmydriatic retinal camera; (FoV) available in 20, 30, and 35	(1) MESSIDOR program, in Saudi Arabia.<br>(2) Bin Rushed Ophthalmic Center, in Saudi Arabia.<br>(3) Magrabi Eye center, in Saudi Arabia.	**Details unavailable**
	Drishti-GS (Sivaswamy et al., 2014)	Glaucoma, non-glaucoma	**Details unavailable**	(1) Aravind eye hospital, in Madurai.	**Details unavailable**
	RIM-ONE-DL (Fumero et al., 2020)	Glaucoma, non-glaucoma	(1) Nidek AFC-210 non-mydriatic fundus camera.<br>(2) A non- mydriatic Kowa WX 3D stereo fundus camera.	(1) Hospital Universitario de Canarias, in Tenerife.<br>(2) Hospital Clinico Universitario San Carlos, in Madrid.<br>(3) Hospital Universitario Miguel Servet, in Zaragoza.	Gradable and non-gradable
	REFUGE (Orlando et al., 2020)	Glaucoma, non-glaucoma	(1) Zeiss Visucam 500 fundus camera.<br>(2) Canon CR-2 device.	(1) From several hospitals and clinical studies from Chinese patients (***details unavailable***).	Gradable and non-gradable
**An instance of RWD**		A spectrum of glaucoma suspect, mild, moderate, and severe glaucoma	(1) Zeiss CIRRUS photo 800.<br>(2) Zeiss FF450 fundus camera.	(1) Illinois Eye and Ear Infirmary	Gradable and non-gradable

3 Data Visualizations via T-SNE

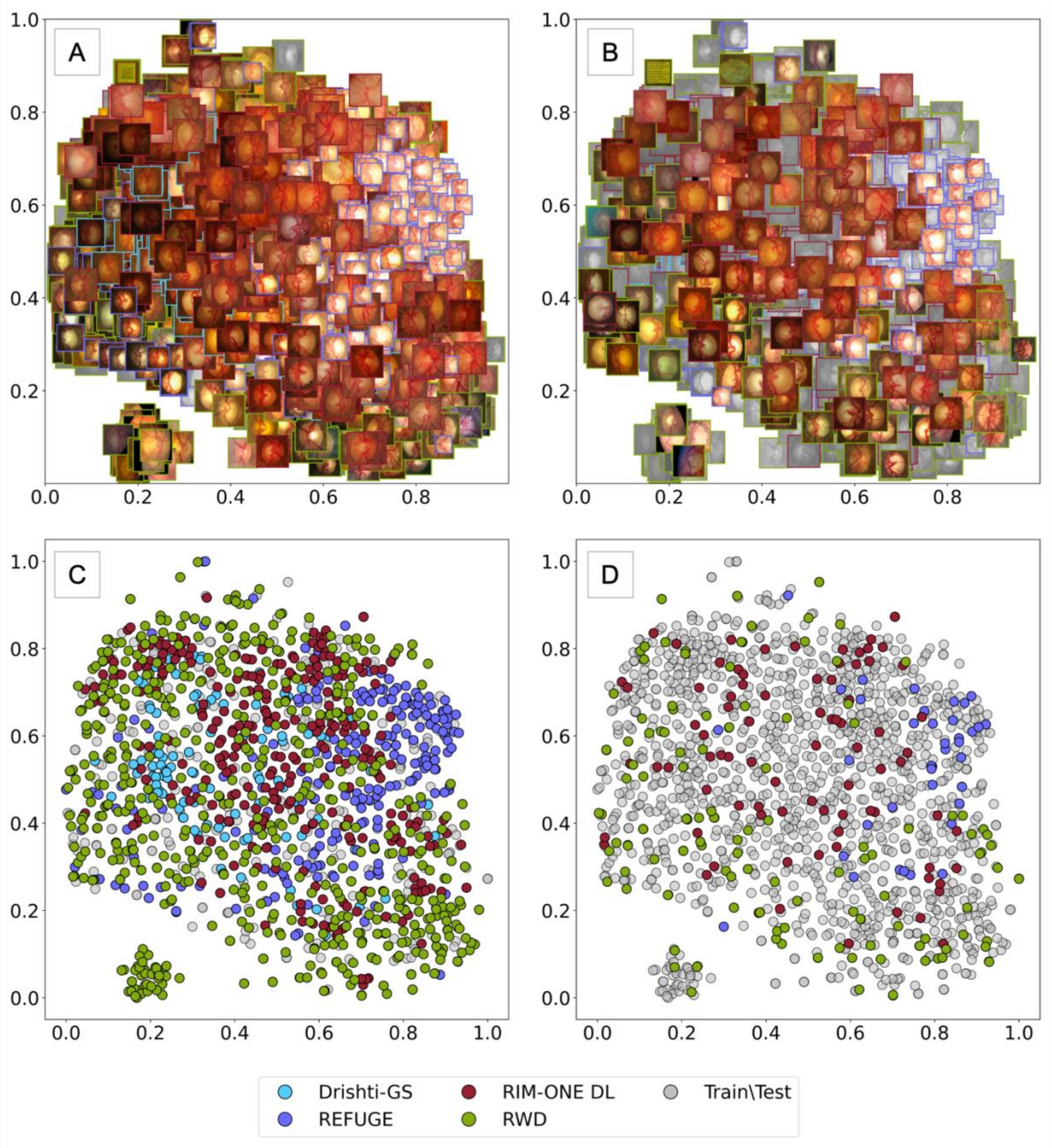

Figure 1. ResNet-50 features visualization for classification data (cropped fundus images) via T-SNE. T-SNE results shown as original input images for (A) train sets, and (B) test sets. T-SNE results shown as scatter points for (C) train sets, and (D) test sets. In parts (A) and (C), the gray color represents test data on the train set plots. Conversely, in parts (B) and (D), the gray color represents train data on the test plots.

Figure 1 shows the T-SNE results for the cropped images in RWD, Drishti-GS, RIM-ONE DL, and REFUGE datasets used in the classification task. Parts (A) and (C) display the T-SNE results for the training datasets, while parts (B) and (D) show the T-SNE results for the test

datasets. In parts (A) and (B), the T-SNE results are visualized using a cropped-view representation of the fundus images, while parts (C) and (D) present the same T-SNE results as scatter plots. In parts (A) and (C), the gray color represents test data on the train set plots. Conversely, in parts (B) and (D), the gray color represents train data on the test plots.

The RWD showed the largest spread in its T-SNE projections as indicated by a trace of 0.146 for its covariance matrix. The other datasets, in order of decreasing trace values, were RIM-ONE DL, REFUGE, and Drishti-GS with covariance traces of 0.085, 0.078, and 0.050 respectively, indicating lower spread in their T-SNE projections. Table 2 shows the feature spread (TSNE covariance trace) and similarity (Wasserstein distance) for the union of public datasets and RWD shown in Figure 1. The trace of the T-SNE covariance matrix for the union of public train and test sets is 0.086 and 0.089, respectively. For the RWD train and test sets, the values are 0.147 and 0.141. The Wasserstein distances for both the public train and test sets and the RWD train and test sets are 0.003, and 0.005, respectively. The Wasserstein distance between the public and RWD train sets is 0.164, and between their test sets is 0.202.

Table 2. Comparison of feature spread (T-SNE covariance trace) and similarity (Wasserstein distance) between the union of public datasets and real-world data (RWD) using off-the-shell ResNet-50 model.

**Metrics**	**Between datasets**				**Within datasets**			
	$\mathrm{Public}_{\mathrm{tr}}$	$\mathrm{RWD}_{\mathrm{tr}}$	$\mathrm{Public}_{\mathrm{te}}$	$\mathrm{RWD}_{\mathrm{te}}$	$\mathrm{Public}_{\mathrm{tr}}$	$\mathrm{Public}_{\mathrm{te}}$	$\mathrm{RWD}_{\mathrm{tr}}$	$\mathrm{RWD}_{\mathrm{te}}$
Feature spread (Covariance trace)	0.086	0.147	0.089	0.141	0.086	0.089	0.147	0.141
Feature similarity (Wasserstein distance)	0.164		0.202		0.003		0.005	

* tr stands for the train set.

* te stands for the test set.

In addition to Table 2 results, generated by a pre-trained off-the-shell ResNet50 model, we summarized similar results when using the public and RWD glaucoma classification models in Table 3. The Wasserstein distance between the feature maps of the public train and test sets using the public model is 0.005. The computed distance for the RWD train and test sets using the RWD model is 0.003. The Wasserstein distance between public and RWD test sets, using public model, is 0.140, and using RWD model is 0.060.Table 3. Comparison of feature spread (TSNE covariance trace) and similarity (Wasserstein distance) between the union of public datasets and real-world data (RWD).

**Metrics**	**Public model**	**RWD model**

	$Public_{te}$	RWD_{te}	$Public_{tr}$	$Public_{te}$	$Public_{te}$	RWD_{te}	RWD_{tr}	RWD_{te}
Feature spread (Covariance trace)	0.131	0.083	0.200	0.131	0.138	0.144	0.148	0.144
Feature similarity (Wasserstein distance)	0.140		0.005		0.060		0.003	

* tr stands for the train set.

* te stands for the test set.

4 IoU Per Test Image in OD Segmentation

To elaborate on the performance of each trained OD segmentation model, we examined the distribution of IoU across test images. Figure 2 (A) shows box plots of IoU per image using public-public, public-RWD, RWD-RWD, and RWD- public models for segmenting OD. We showed the mean and median IoU per model by a red and a blue line per box plot, respectively. As shown in Figure 2 (A), we found that the public model has the lowest IoU variability ($SD = 0.04$) for segmenting OD on the public data, but this variability increased by 350% when we tested the model on RWD ($SD = 0.18$). Since the public data is homogeneous, the public model performs nearly identical across public test data resulting in a low IoU variability. In contrast, since RWD is heterogeneous, the public model does not have an identical performance across the RWD test set resulting in a high IoU variability. Similarly, the IoU variability of the RWD model from testing it on public data to RWD increases by 28% from $SD = 0.07$ to $SD = 0.09$. The increment in IoU variability from public data to RWD using either public or RWD trained model shows that the RWD is more challenging than public data.

Further, in Figure 2 (B), and (C), in each row, we visualized the original image, ground truth OD mask, and predicted OD mask when the IoU for an image is less than 0.5 to identify challenging images in public-RWD, public-public, RWD-RWD, and RWD-public experiments, respectively. We found that all images in the public-public experiment have an IoU greater than 0.5. As indicated in Figure 2 (B), we found that images with multiple bright spots, small, or dark OD regions in the RWD test set are challenging for the public model to predict accurately (IoU < 0.5). In contrast, as indicated in Figure 2 (C), the RWD model results in an IoU less than 0.5 for solely 0.02% of images in the RWD test set.

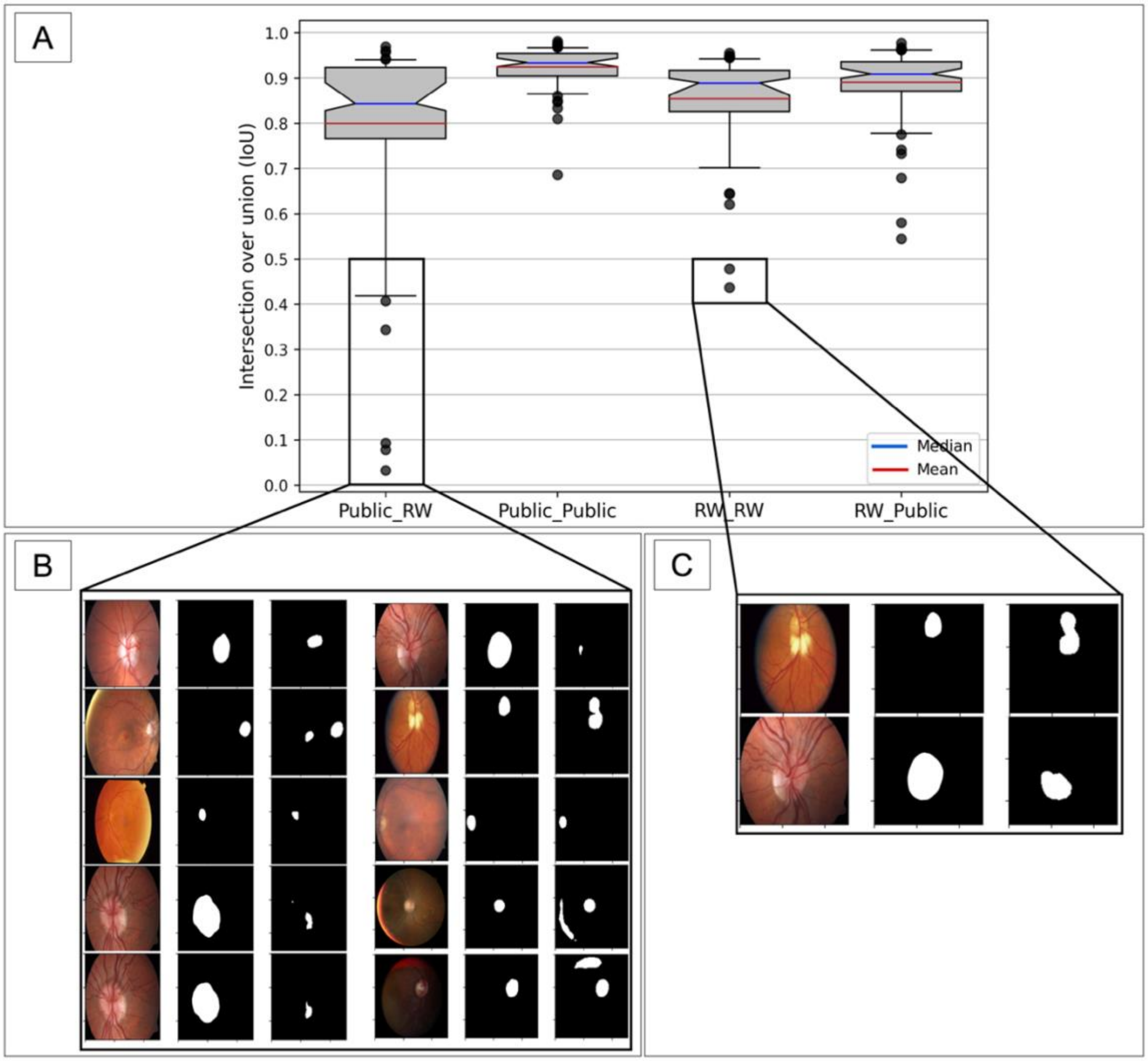

Figure 2. Inspection of Intersection over Union (IoU) for public-public, RWD-RWD, public-RWD, and RWD-public. (A) Box plots of IoU per test image for Public-RWD, Public-Public, RWD-RWD, and RWD-Public experiments. (B), (C) Challenging images that resulted in low OD segmentation performance. Each row respectively contains original images, grand truth masks, and predicted masks with an IoU < 0.5.

5 Glaucoma Classification

Error! Reference source not found. (A)-(F) shows the ROC plots for RWD-RWD, public-RWD, (RWD+public)-RWD, RWD-public, public-public, and (RWD+public)-public models, respectively in blue. The shaded blue area around ROC shows 95% confidence interval and the red straight line shows the performance of a random classifier. As the Area Under the ROC (AUROC) is higher, the ability of a model in discriminating between classes (e.g., glaucoma vs. non-glaucoma) is higher. We showed that public-public model has the highest AUROC value (mean AUROC [95% CI] = 95% [94%, 97%]) among experiments, as indicated in **Error! Reference source not found.** (E). However, its AUROC majorly shrinks (mean AUROC [95% CI] = 76 [74, 80]) when tested on RWD, as shown in **Error! Reference source not found.** (B). On the other hand, ROC for the model trained on RWD, is stable when tested on either RWD or public data,

as shown in **Error! Reference source not found.** (A) and (D); it achieves similar AUROC values across datasets (mean AUROC [95% CI] on RWD = 86 [84, 88], mean AUROC [95% CI] on public = 86 [85, 88]).

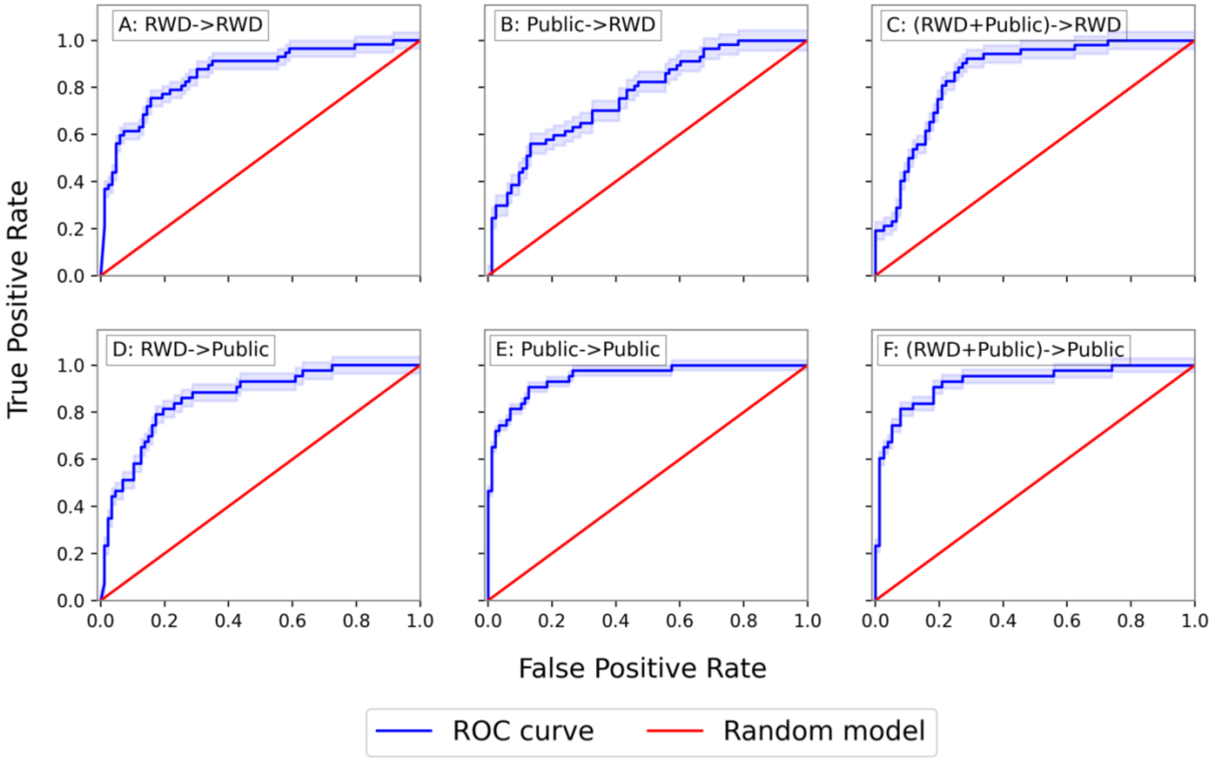

Figure 3. Area Under the Receiver Operating Characteristic Curve (AUROC) for (A) RWD-RWD model, (B) Public-RWD model, (C) (RWD+Public)-RWD model, (D) RWD-Public model, (E) Public-Public model, and (F) (RWD+Public)-Public model.

5.1 Generalization on A Larger Sample

We further tested the RWD and public-trained glaucoma classification models on a larger unseen sample of our RWD consisting of $5{,}275$ images. We summarized the comparison results for public versus RWD-trained models on the $5{,}275$ images in Table 4. As indicated, the RWD-trained model outperforms the public-trained model by 15% higher accuracy, showing that it generalizes majorly better to the $\sim 5.2K$ unseen RWD. Similarly, the RWD-trained model achieves higher SEN, PPV, and F_1 score when tested on $\sim 5.2K$ unseen RWD than the public-trained model.

Table 4. Comparison results of public-versus Real-World Data (RWD)-trained models tested on 5,275 unseen samples of our RWD for glaucoma classification.

**Dataset**		**Evaluation metrics on test set**			
**Train**	**Test**	**Acc**	**SEN**	**PPV**	**F_1 score**
Public	RWD-5.2K	0.63	0.26	0.52	0.34
RWD		0.78	0.45	0.54	0.48

5.2 T-SNE Visualization

In Figure 4 (A)-(D), we showed the 2D projection of learned features by public-public, RWD-RWD, public-RWD, and RWD-public models, respectively. TSNE results in parts (A) and (C) of Figure 4 are generated with public model and the TSNE results in parts (B) and (D) are generated with RWD model. Projected glaucoma and non-glaucoma features per test image are shown as red circles and blue circles, respectively. We found that there is a clear cluster pattern between glaucoma and non-glaucoma features when we used the public-trained model on the public test data, as shown in Figure 4 (A). However, this cluster pattern disappeared when we tested the public-trained model on RWD, as shown in Figure 4 (C). Therefore, our results suggest that while the public-trained model can clearly discriminate between glaucoma and non-glaucoma images in public test data, it cannot maintain its performance on the RWD. In contrast, based on Figure 4 (B) and (D), the RWD-trained model reveals a similar pattern in discriminating between glaucoma and non-glaucoma images across datasets (public, RWD).

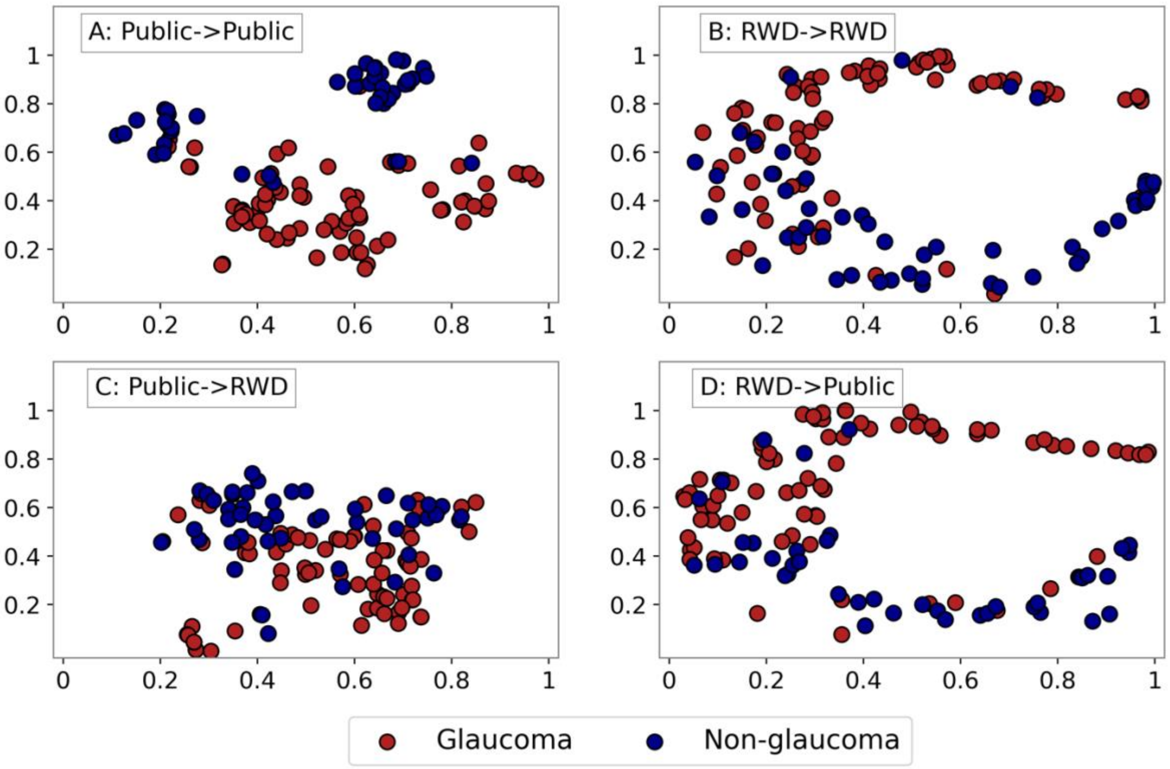

Figure 4. T-SNE visualization of glaucoma versus non-glaucoma representations per test image using (A) public-public, (B) RWD-RWD, (C) public-RWD, and (D) RWD-public models.
